# Impact of comorbid depression on clinical outcomes in patients with peripheral artery disease: a systematic review and meta-analysis

**DOI:** 10.3389/fcvm.2026.1854607

**Published:** 2026-08-21

**Authors:** Chuwen Chen, Lijia Wei, Jing Hu, Huanrui Hu, Bin Huang, Jichun Zhao, Wayne W. Zhang, Xiyang Chen

**Affiliations:** 1 Division of Vascular Surgery, Department of General Surgery, West China Hospital, Sichuan University, ChengDu, China; 2 Division of Health Management Centre, West China Fourth Hospital Sichuan University, ChengDu, China; 3 Cardiovascular Research Lab, Chinese Institutes for Medical Research, BeiJing, China; 4 Department of Vascular Surgery, Anzhen Hospital, Capital Medical University, Beijing, China; 5 Division of Vascular and Endovascular Surgery, University of Washington, Seattle, WA, United States

**Keywords:** amputation, depression, major adverse cardiovascular events, major adverse limb events, mortality, peripheral artery disease

## Abstract

**Background:**

Depression is a common comorbidity in cardiovascular disease; however, its prognostic impact in peripheral artery disease (PAD) remains uncertain. This study evaluated the association between depression and clinical outcomes in patients with PAD.

**Methods:**

PubMed, Embase, Web of Science, Cochrane Library, and ClinicalTrials.gov were systematically searched from inception to December 2025. Observational studies comparing PAD patients with and without depression were included. Primary outcomes included all-cause mortality, major adverse cardiovascular events (MACE), major amputation, and major adverse limb events (MALE). Random-effects meta-analyses were performed.

**Results:**

Thirteen studies involving 681,959 patients were included. Compared with those without depression, PAD patients with depression were generally younger and had a higher burden of cardiovascular and metabolic comorbidities. Depression was not significantly associated with all-cause mortality in pooled analyses of either odds ratios (OR = 2.01; 95% CI: 0.77–5.27; *p* = 0.15) or hazard ratios (HR = 1.15; 95% CI: 0.97–1.36; *p* = 0.11). However, depression was significantly associated with worse limb-related outcomes, including major amputation (OR = 1.25; 95% CI: 1.15–1.37; *p* < 0.001, HR = 1.36; 95% CI: 1.21–1.54; *p* < 0.001) and MALE (OR = 1.24, 95% CI: 1.16–1.33, *p* < 0.001). Depression was also associated with a higher risk of MACE in both pooled OR analysis (OR = 1.82; 95% CI: 1.02–3.26; *p* = 0.04) and time-to-event analysis (HR = 1.24; 95% CI: 1.15–1.35; *p* < 0.001).

**Conclusions:**

In PAD, comorbid depression was associated with higher MALE and MACE risks, but not all-cause mortality. These findings suggest that mental health assessment may be considered as part of comprehensive PAD care.

## Introduction

Depression is one of the most common mental disorders worldwide and a major contributor to disability, reduced quality of life, and premature mortality (1). Beyond its psychological burden, accumulating evidence suggests that depression is closely associated with a range of adverse physical health outcomes, particularly among patients with cardiovascular disease. Previous studies have shown that depression is not only an independent risk factor for coronary artery disease but also significantly associated with increased hospitalization, poor functional recovery, recurrent cardiovascular events, and a higher risk of death (2, 3). These findings suggest that depression may play an important role in the development, progression, and prognosis of vascular disease.

Peripheral artery disease (PAD), the lower-extremity manifestation of systemic atherosclerosis, is one of the most common vascular diseases and affects more than 200 million people worldwide (4, 5). PAD is associated with a poor prognosis, with an estimated 5-year mortality rate of 82.4 per 1,000 patients (5, 6). It can lead to severe limb-related complications, including intermittent claudication, chronic limb-threatening ischemia, and amputation, while also substantially increasing the risks of major adverse cardiovascular events (MACE) and mortality. Approximately half of PAD-related hospitalizations are attributable to limb complications, whereas the remainder are related to major adverse cardiovascular events (5, 7). Established risk factors for PAD include smoking, hypertension, hypercholesterolemia, diabetes mellitus, overweight, male sex, and older age (8).

Given the chronic course of PAD, its marked functional limitations, substantial pain burden, and generally poor prognosis, depression may be of particular clinical relevance in this population (5). Depression is a relatively common comorbidity among patients with PAD and may further worsen clinical outcomes by adversely affecting treatment adherence, functional recovery, and disease progression. Although the role of depression in coronary artery disease has been more clearly established, its relationship with PAD remains insufficiently understood (2, 3). Some studies suggested that depressive symptoms are associated with PAD and may increase the risk of hospitalization (9, 10). However, although a growing body of research has examined various clinical outcomes in patients with PAD, previous studies have largely focused on quality of life, temporal changes in depressive symptoms, revascularization strategies, traditional risk factors, and epidemiological characteristics. Evidence specifically addressing the association between depression and subsequent clinical outcomes in PAD remains heterogeneous and has not been comprehensively synthesized (9, 11). Therefore, we conducted an updated meta-analysis to evaluate the association between depression and clinical outcomes in patients with PAD, intending to inform clinical management and improve care for this vulnerable population.

## Methods

This study was a systematic review and meta-analysis of previously published data and did not involve human participants or identifiable personal information. Therefore, ethical approval and informed consent were not required. The study protocol was registered in PROSPERO (CRD420251126659), and the review was conducted in accordance with the Preferred Reporting Items for Systematic Reviews and Meta-Analyses (PRISMA) guidelines (12).

### Search strategy and literature screening

Two independent investigators (CC and LW) systematically searched PubMed, Embase, Web of Science, ClinicalTrials.gov, the World Health Organization International Clinical Trials Registry Platform, and Cochrane Library databases from inception to December 2025. To minimize the risk of missing relevant studies, the search strategy incorporated both controlled vocabulary terms, including Medical Subject Headings (MeSH) in PubMed and Emtree terms in Embase, and free-text keywords. For peripheral artery disease, the search terms included “peripheral arterial disease,” “peripheral vascular disease,” “PAD,” “lower extremity arterial disease,” “intermittent claudication,” “critical limb ischemia,” and related synonyms. For depression, a comprehensive range of terms was used to capture both diagnostic and symptom-based definitions, including “depression,” “depressive disorder,” “major depressive disorder,” dysthymia, depressive symptoms, depressed, and related terms. Boolean operators (“OR” and “AND”) were used to combine disease-related and outcome-related terms. Search strategies were adapted for each database to account for differences in indexing systems between MeSH and Emtree. In addition, the reference lists of relevant studies and reviews were manually screened to identify potentially eligible articles. Any discrepancies during the literature selection process were resolved through discussion with a third investigator (JH) until consensus was reached.

### Inclusion and exclusion criteria

#### Inclusion criteria

Studies were included if they met the following criteria: (1) participants had PAD, defined by clinical diagnosis, imaging findings, ankle-brachial index measurement, or administrative diagnostic codes (e.g., ICD-9/10-CM codes); (2) the PAD phenotype or clinical presentation included lower extremity arterial disease, intermittent claudication, or critical limb ischemia; (3) depression was defined by clinical diagnosis, ICD-9/10-CM codes, or validated depression scales with established cutoff values (e.g., PHQ-9, GDS-15, HADS-D, GAD-7); (4) the study was an original study with an observational design, including cohort, case–control, or cross-sectional studies, and examined the association between depression and clinical outcomes in patients with PAD; (5) the study reported at least one outcome of interest, including death, MACE, major amputation, or major adverse limb events (MALE); and (6) the full-text article was published in English.

#### Exclusion criteria

Studies were excluded if they met any of the following criteria: (1) pediatric or non-human studies; (2) case reports, case series, reviews, editorials, conference abstracts, or studies without original data; (3) depression assessed using non-validated instruments or unclear diagnostic criteria; (4) insufficient data to estimate effect sizes or confidence intervals; and (5) duplicate publications or overlapping datasets, in which case only the most comprehensive study was included.

### Outcome definitions

The primary outcomes included all-cause mortality, MALE, and MACE. MALE was defined as a composite of major amputation, minor amputation, and any amputation, or amputation plus repeat peripheral vascular intervention. MACE was defined as a composite of cardiovascular death, myocardial infarction, and stroke.

### Data extraction and quality assessment

#### Data extraction

Two reviewers (BH and HH) independently extracted data on patient baseline characteristics, procedural outcomes, and clinical outcomes from the included articles. In cases of uncertainty or disagreement, a third reviewer (XC) was consulted to review the study and reach a consensus. Clinically relevant variables, including crude event numbers (when available), event rates for each cohort, type and value of effect estimate, confidence intervals, depression severity, antidepressant use, and follow-up duration, were extracted when available. Adjusted hazard ratio/unadjusted hazard ratios were extracted directly from articles when reported or estimated from Kaplan–Meier curves using Engauge Digitizer software (13).

#### Quality assessment

Study quality was independently assessed by two reviewers using design-specific tools. The Newcastle–Ottawa Scale (NOS) was applied to observational studies (14), including retrospective and prospective cohort studies, whereas randomized controlled trials (RCTs) were evaluated using the Cochrane Risk of Bias tool. Disagreements were resolved by consensus, with third-party adjudication when necessary. Observational studies with an NOS score ≥7 and RCTs assessed as having a low risk of bias across all domains were considered to be of high methodological quality. Given the observational design of the included studies, potential sources of bias, especially residual confounding and variability in depression assessment methods, were anticipated and addressed using random-effects meta-analysis and by interpreting the results with appropriate caution.

### Statistical analysis

Data analyses were performed using Review Manager (Rev Man) Version 5.4 (The Cochrane Collaboration, 2020). Continuous variables are presented as means ± standard deviations, and categorical variables are presented as percentages. When necessary, medians and ranges were converted to means and standard deviations using the method reported by Wan et al. (15). Publication bias was assessed using funnel plots when at least 10 studies were available. Statistical heterogeneity was evaluated using Cochran's *Q* test and quantified with the *I*^2^ statistic, with *I*^2^ values >50% indicating substantial heterogeneity. A random-effects model was applied in the presence of significant heterogeneity. Sensitivity analysis and prediction intervals (PIs) were reported as described (16). We set the *p*-value ≤0.05 as the level of statistical significance.

## Results

### Characteristics of the included studies and patient demographics

**Figure 1:**
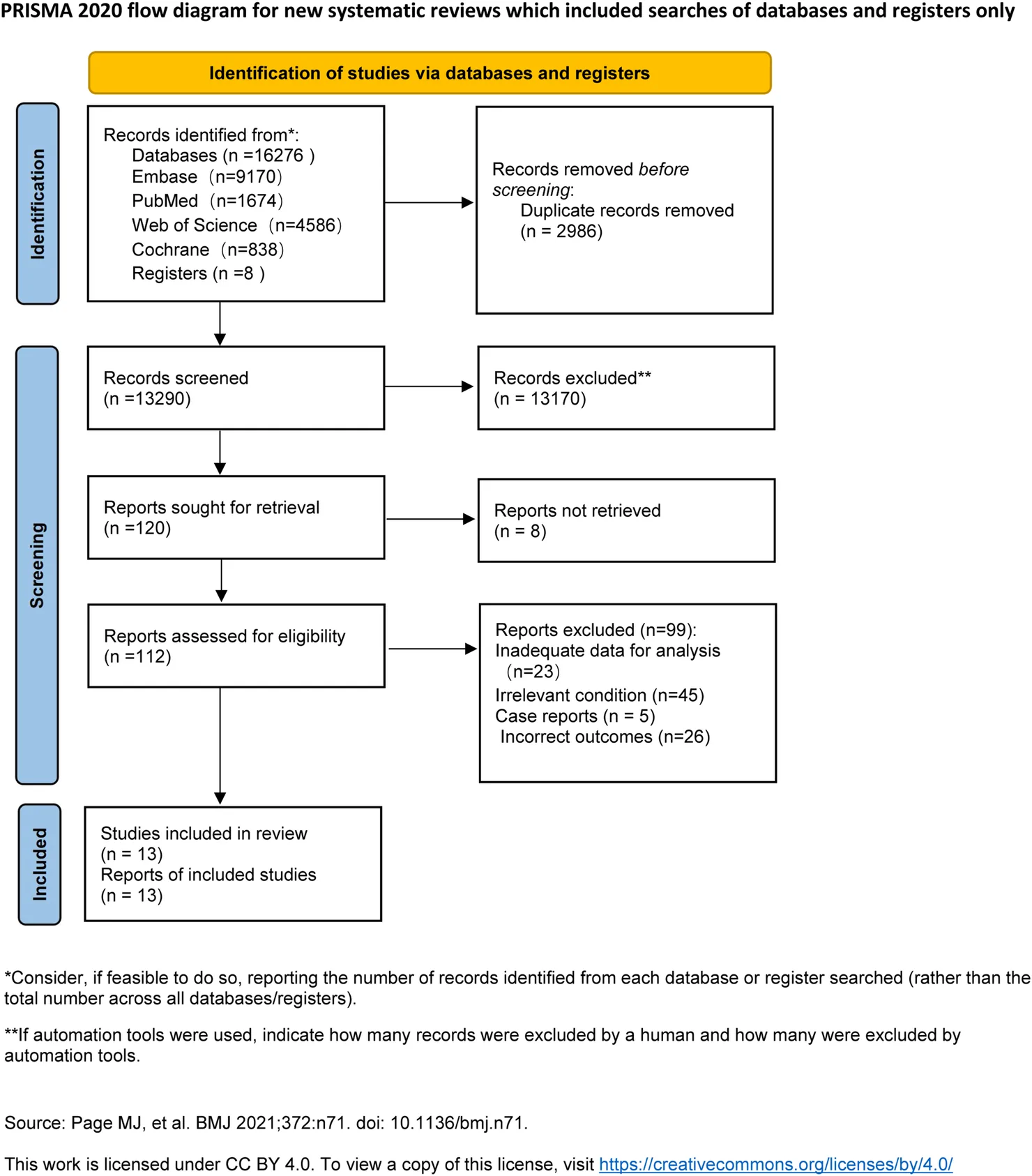
Flowchart of this analysis protocol. PRISMA, Preferred Reporting Items for Systematic Reviews and Meta-Analyses.

For the quantitative synthesis, we included 13 studies, comprising 11 retrospective cohort studies (17–27), 1 observational cohort (28), and one prospective cohort study (29). The NOS scores of the included non-randomized studies are presented in Table 1, and all 13 studies were considered to be of high methodological quality (NOS score ≥7). Figure 1 illustrates the study selection process, and Table 1 summarizes the characteristics of the 13 included studies.

**Table 1:** Characteristics of the included studies.

Study (year)	Study interval	Country	Study type	Depression	No depression	Depression assessment	Outcomes	Follow-up time	NOS
Shalaeva et al. (2024) (29)	2022–2023	Switzerland	Prospective	331	454	Patient Health Questionnaire- 9	One-year all-cause mortality	1 year	8
Schmittling (2025) (21)	2020–2021	USA	Retrospective	3,574	15,869	ICD-10-CM	In-hospital mortality; length of hospital stay	In-hospital period	7
Koksoy et al. (2025) (24)	Jan. 20231–Dec. 2023	USA	Retrospective	22	79	ICD-9-CM//GAD-7	Major amputation; all-cause mortality	1 year	7
Harris et al. (2023) (20)	2011–2017	USA	Retrospective	37,089	347,340	ICD-9-CM/ICD-10-CM	In-hospital; major amputation; mortality	In-hospital period	8
Suarez et al. (2023) (27)	2010–2019	USA	Retrospective	169	169	ICD-9/10 codes or antidepressant use	Reintervention; major amputation; death	2 years	7
Zielke et al. (2024) (19)	2007–2018	USA	Retrospective	212	2,775	ICD-9-CM/ICD-10-CM	Major amputation; all-cause mortality	Median 17.4 months	8
Arya et al. (2018) (18)	2003–2014	USA	Retrospective	24,895	130,752	ICD-9-CM	Major amputation; all-cause mortality	All patients: median 5.9 years	7
Cherr et al. (2007) (23)	2000–2004	USA	Retrospective	78	138	General Health Questionnaire-12 with score >3	Primary patency; assisted primary patency; major amputation	All patients: mean 23.4 ± 14.1 months	6
						2. Further evaluation by a physician			
Cherr et al. (2008) (25)	2000–2005	USA	Retrospective	90	167	General Health Questionnaire-12 with score >3	Mortality; MACE; non-home discharge	All patients: mean 28.3 ± 16.2 months	7
						2. Further evaluation by a physician			
McDermott et al. (2016) (28)	1998–2014	USA	Observational cohort	186	765	Geriatric Depression Scale-15	All-cause mortality;	Participants were followed up until death	7
Ramirez et al. (2021) (26)	2012–2014	USA	Retrospective	5,472	59,345	ICD-9-CM	Amputation (major and minor); non-home discharge; length of stay; postoperative complications	In hospital course	7
Smolderen et al. (2018) (22)	2005–2010	USA	Retrospective	265	532	ICD-9-CM	Mortality; major amputation; MACE	All patients: median 955 days	8
Zahner et al. (2020) (17)	2012–2013	USA	Retrospective	10,512	105,496	ICD-9-CM	Major amputation; length of stay; inpatient mortality; postoperative complications	In hospital course	7

NOS, Newcastle–Ottawa Scale; ICD-10-CM, International Classification of Diseases, 10th revision, clinical modification; ICD-9-CM, International Classification of Diseases, 9th revision, clinical modification; PHQ-9, Patient Health Questionnaire-9; GAD-7, Generalized Anxiety Disorder-7; MACE, major adverse cardiovascular events.

Across the 13 studies (17–29), a total of 681,959 individuals were included. Patients with depression were significantly younger than their non-depressed counterparts [mean difference (MD) = 2.71 years; 95% CI: 2.07–3.35; *p* < 0.001; *I*^2^ = 96%; Supplementary Figure 1]. In addition, compared with patients without depression, those with depression had a higher prevalence of coronary artery disease (OR = 1.17; 95% CI: 1.13–1.20; *p* < 0.001; *I*^2^ = 20%; Table 2), diabetes (OR = 1.17; 95% CI: 1.09–1.26; *p* < 0.001; *I*^2^ = 87%; Table 2), dyslipidemia (OR = 1.25; 95% CI: 1.05–1.50; *p* < 0.001; *I*^2^ = 97%; Table 2), heart failure (OR = 1.29; 95% CI: 1.19–1.40; *p* < 0.001; *I*^2^ = 77%; Table 2), obesity (OR = 1.44; 95% CI: 1.24–1.60; *p* < 0.001; *I*^2^ = 88%; Table 2), pulmonary disease (OR = 1.39; 95% CI: 1.24–1.55; *p* < 0.001; *I*^2^ = 89%; Table 2), and renal disease (OR = 1.11; 95% CI: 1.00–1.23; *p* *=* 0.05; *I*^2^ = 91%; Table 2) and a lower proportion of males (OR = 0.65; 95% CI: 0.59–0.72; *p* < 0.001; *I*^2^ = 93%; Table 2). There were no significant differences between groups in body mass index (MD = 0.43 kg/m^2^; 95% CI: −0.10 to 0.96; *p* = 0.11; *I*^2^ = 66%) and hypertension (OR = 1.08; 95% CI: 0.98–1.12; *p* = 0.11; *I*^2^ = 87%; Table 2).

**Table 2:** Pooled patient baseline characteristics.

Variables	No. of studies	No. of patients	Depression	No-depression	Odds ratio, M-H random, 95% CI	*p*-Value	*I*^2^ (%)
Coronary artery disease (8, 16–18, 20–22, 24, 26)	9	545,557	63,151	482,406	1.17 (1.13–1.20)	**<0** **.** **001**	20
Diabetes (8, 15–18, 20–26)	12	727,333	79,321	648,012	1.17 (1.09–1.26)	**<0** **.** **001**	87
Hyperlipidemia (15–18, 20–22, 24)	8	545,219	62,982	482,237	1.25 (1.04–1.50)	**0** **.** **02**	97
Gender (male) (15–21, 23–26)	11	746,303	82,727	663,576	0.65 (0.59–0.72)	**<0** **.** **001**	93
Heart failure (15–18, 20, 21, 23–26)	10	726,860	79,153	647,707	1.29 (1.19–1.40)	**<0** **.** **001**	77
Hypertension (8, 15–18, 20–24, 26)	11	726,382	79,135	647,247	1.08 (0.98–1.12)	0.11	87
BMI (16, 20, 21, 25, 26)	5	158,281	25,699	132,582	0.43 (−0.1 to 0.96)	0.11	66
Obesity (15, 18, 20, 23, 26)	5	566,836	53,669	513,167	1.44 (1.24–1.60)	**<0** **.** **001**	88
Pulmonary disease (8, 16–18, 20–25)	10	610,540	68,478	542,062	1.39 (1.24–1.55)	**<0** **.** **001**	89
Renal disease (8, 16–18, 20–26)	11	726,382	79,135	647,247	1.11 (1.00–1.23)	**0** **.** **05**	91
Smoke (current/previous) (8, 15–18, 20, 21, 23–26)	11	726,995	79,152	647,843	1.27 (1.22–1.32)	**<0** **.** **001**	59

Bold values indicate statistical significance (*p* ≤0.05).

Note: the bracket value should be: *p* less than or Equal to 0.05

CI, confidence interval; BMI, body mass index.

### All-cause mortality

**Figure 2:**
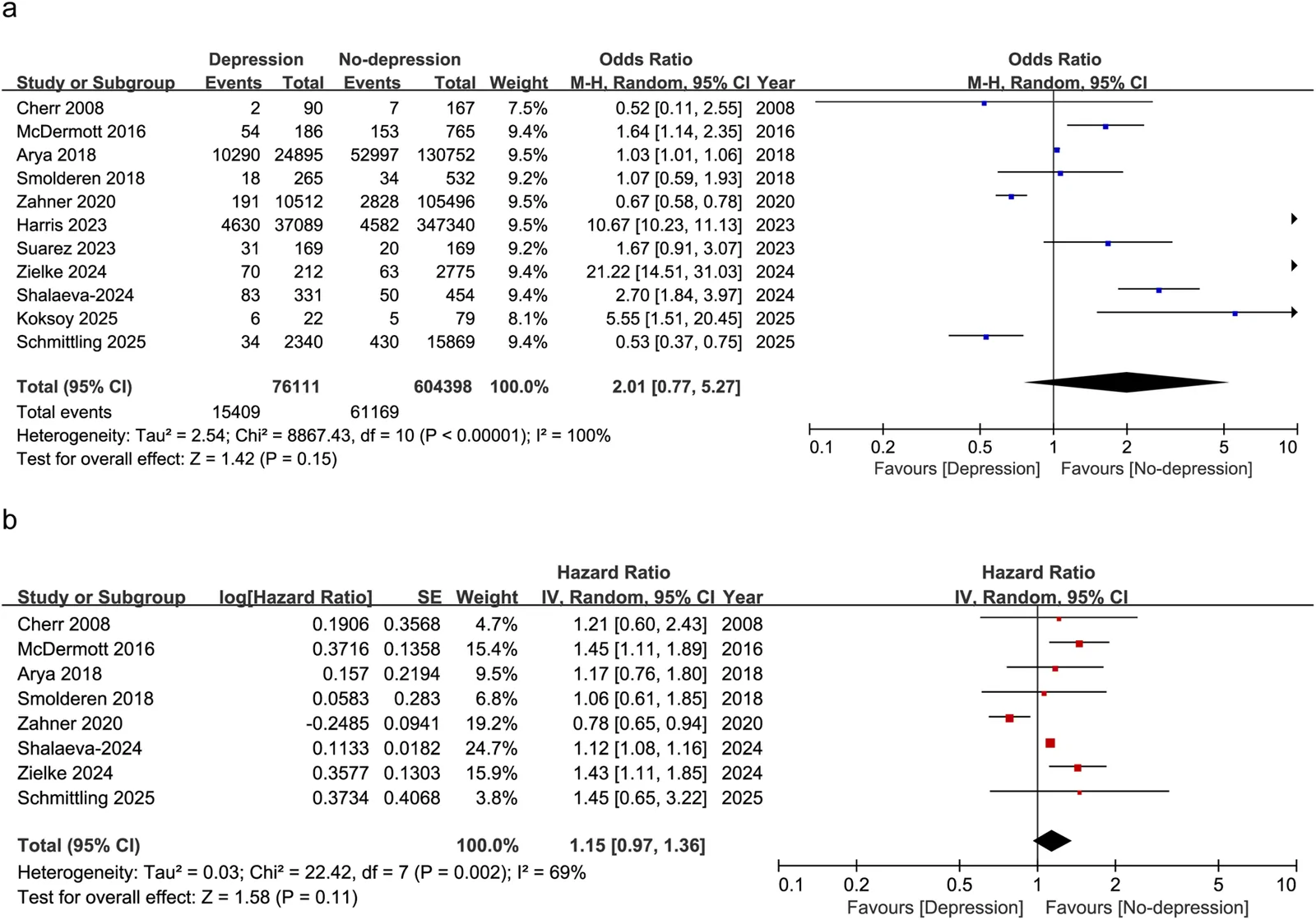
**(a)** Forest plot of the meta-analysis of all-cause mortality between depressed and non-depressed patients. **(b)** Forest plot showing the pooled HR for Kaplan–Meier curves. HR, hazard ratios; M-H, Mantel–Haenszel; CI, confidence interval; SE, standard error.

Eleven studies (17–22, 24, 25, 27–29) reported data on all-cause mortality in the depression group and the no-depression group. The pooled analysis showed no statistically significant difference in all-cause mortality between patients with and without depression (depression vs. no-depression: 20.25% vs. 10.12%; OR = 2.01; 95% CI: 0.77–5.27; *p* = 0.15; *I*^2^ = 100%; 95% PI: −0.96 to 5.48; Figure 2A). To further elucidate the association between depression and prognosis in patients with PAD, we identified eight studies (17–19, 21, 22, 25, 28, 29) that reported 5-year Kaplan–Meier curves or mortality rate data. The pooled time-to-event analysis also showed no statistically significant difference in all-cause mortality risk between the depression and no-depression groups (HR = 1.15; 95% CI: 0.97–1.36; *p* = 0.11; Figure 2B) with moderate heterogeneity (*I*^2^ = 69%).

### Limb-specific outcomes

**Figure 3:**
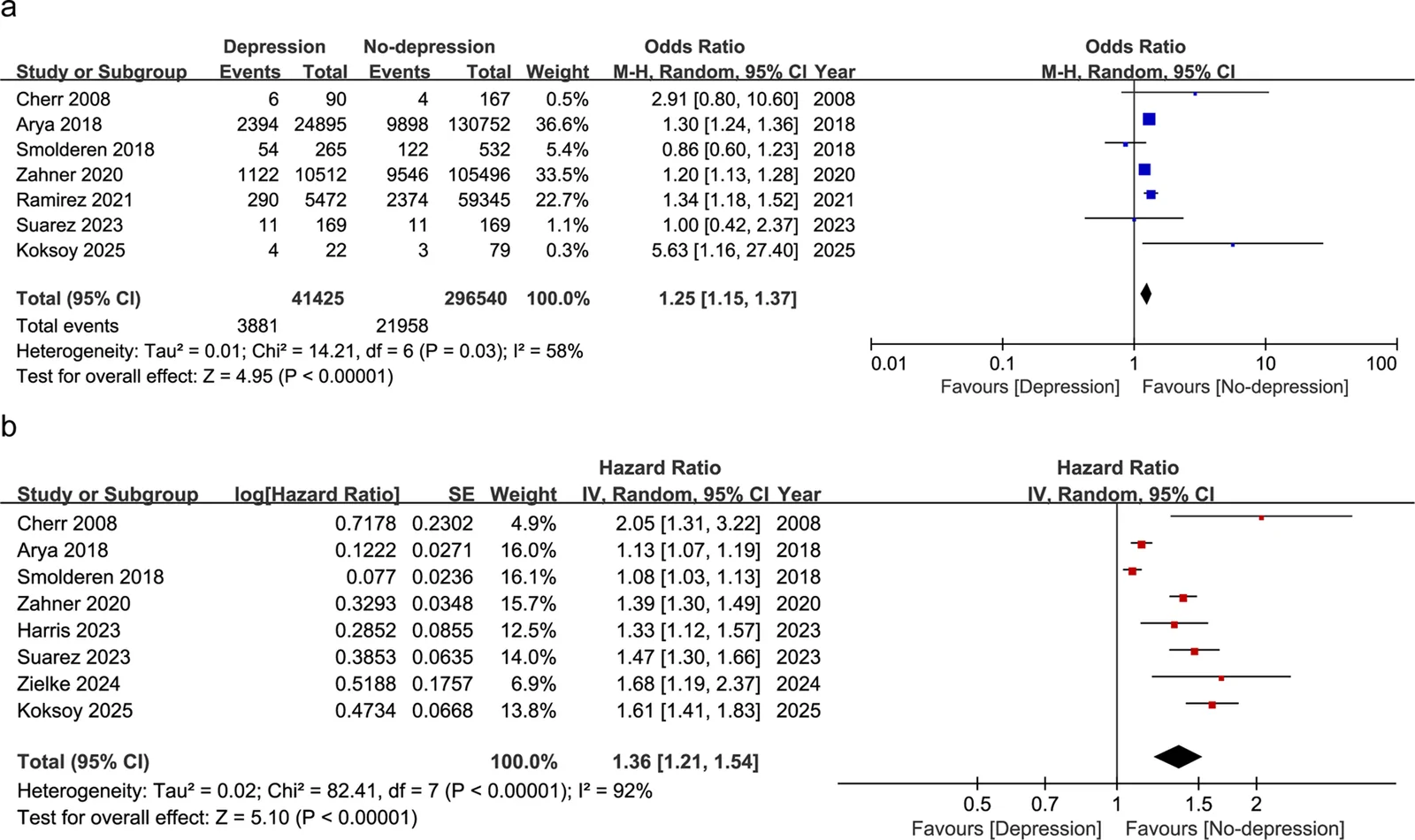
**(a)** Forest plot of the meta-analysis of major amputation between depressed and non-depressed patients. **(b)** Forest plot showing the pooled HR for Kaplan–Meier curves. HR, hazard ratios; M-H, Mantel–Haenszel; CI, confidence interval; SE, standard error.

**Figure 4:**
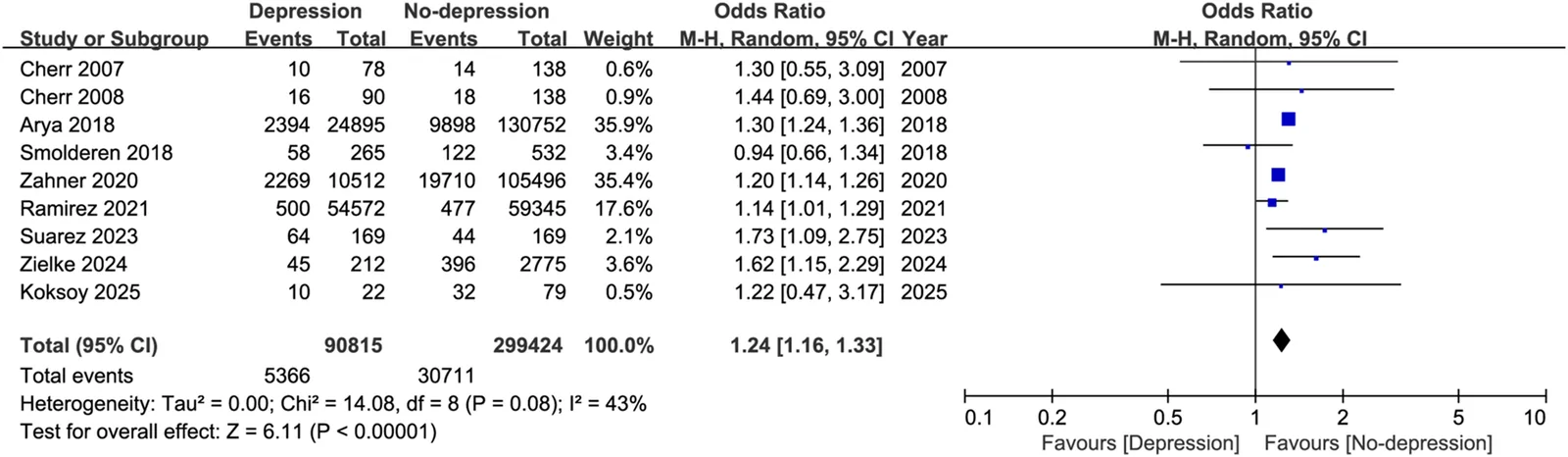
Forest plot of the meta-analysis of MALE between depressed and non-depressed patients. M-H, Mantel–Haenszel; CI, confidence interval; MALE, major adverse limb events.

The analysis of major amputation included seven studies (17, 18, 22, 24–27) involving 41,425 patients with depression and 296,540 patients without depression. The pooled analysis indicated that depression was associated with a higher risk of major amputation (OR = 1.25; 95% CI: 1.15–1.37; *p* < 0.001; *I*^2^ = 58%; 95% PI: 1.00–1.47; Figure 3A). To further clarify the association between depression and major amputation in patients with PAD, we identified eight studies (17–20, 22, 24, 25, 27) that reported 5-year Kaplan–Meier curves or time-to-event data for major amputation. The pooled analysis showed that the risk of major amputation was significantly higher in the depression group than in the no-depression group (depression vs. no-depression: HR = 1.36; 95% CI: 1.21–1.54; *p* < 0.001; Figure 3B), with high heterogeneity (*I*^2^ = 92%). We further analyzed the association between depression and MALE in patients with PAD. The analysis of MALE included nine studies (17–19, 22–27) involving 90,815 patients with depression and 299,424 patients without depression. The pooled analysis showed that depression was associated with a higher risk of MALE (OR = 1.24; 95% CI: 1.16–1.33; *p* < 0.001; *I*^2^ = 43%; 95% PI: 1.05–1.40; Figure 4). As long-term MALE outcomes were not available in the included studies, we were unable to further evaluate the relationship between depression and long-term MALE.

### Major adverse cardiovascular events

**Figure 5:**
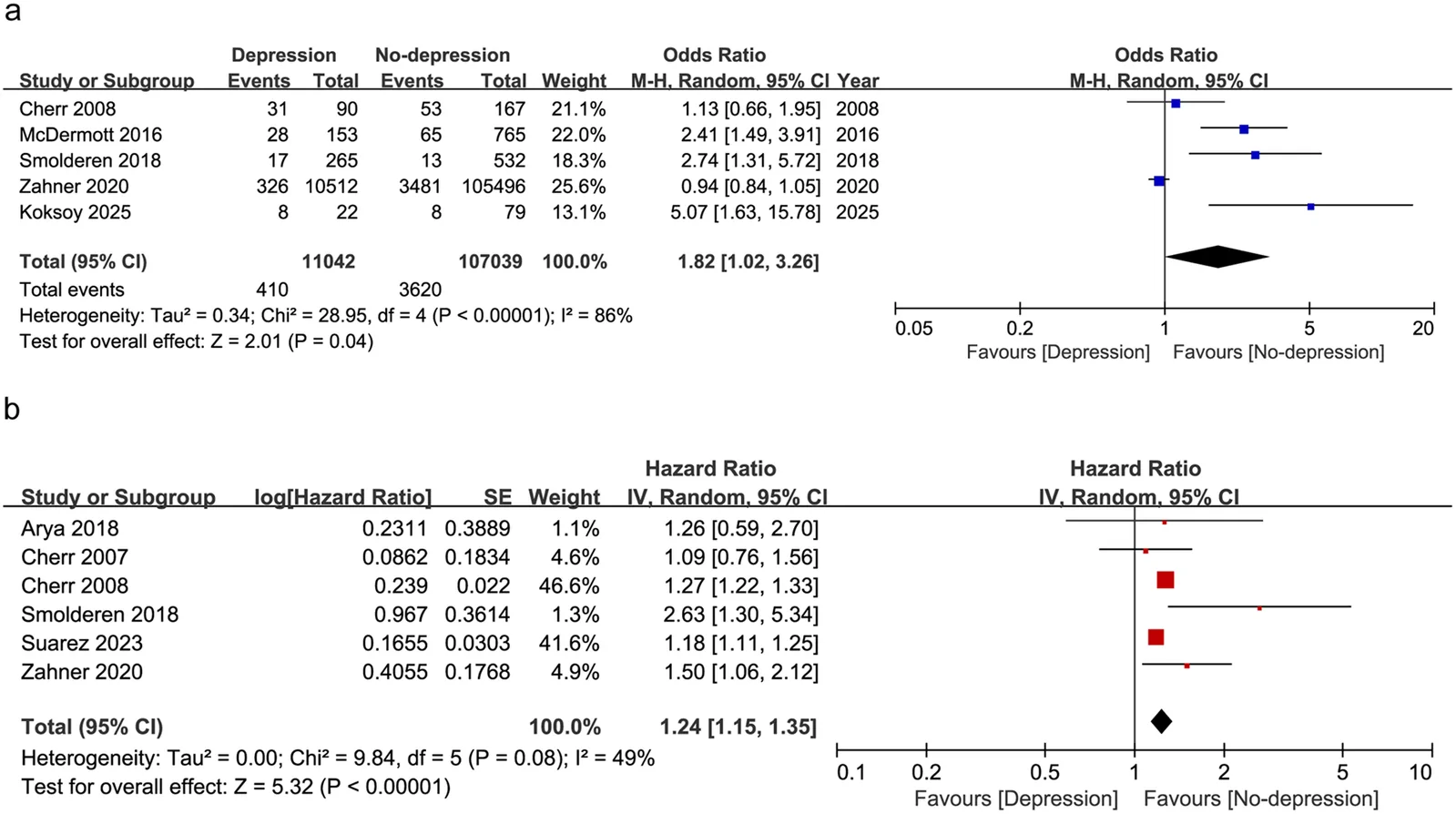
**(a)** Forest plot of the meta-analysis of MACE between depressed and non-depressed patients. **(b)** Forest plot showing the pooled HR for Kaplan–Meier curves. HR, hazard ratios; M-H, Mantel–Haenszel; CI, confidence interval; SE, standard error; MACE, major adverse cardiovascular events.

The analysis of MACE included five studies (17, 22, 24, 25, 28) involving 11,042 patients with depression and 107,039 patients without depression. The pooled analysis demonstrated that depression was associated with a higher risk of MACE (OR = 1.82; 95% CI: 1.02–3.26; *p* = 0.04; *I*^2^ = 86%; 95% PI: −0.44 to 3.26; Figure 5A). To further elucidate the association between depression and MACE in patients with PAD, we identified six studies (17, 18, 22, 23, 25, 27) that reported 5-year Kaplan–Meier curves or MACE rate data. The time-to-event analysis showed that the risk of MACE was significantly higher in patients with depression than in those without depression (HR = 1.24; 95% CI: 1.15–1.35; *p* < 0.001; Figure 5B), with lower heterogeneity (*I*^2^ = 49%).

### Publication bias and sensitivity analysis

**Figure 6:**
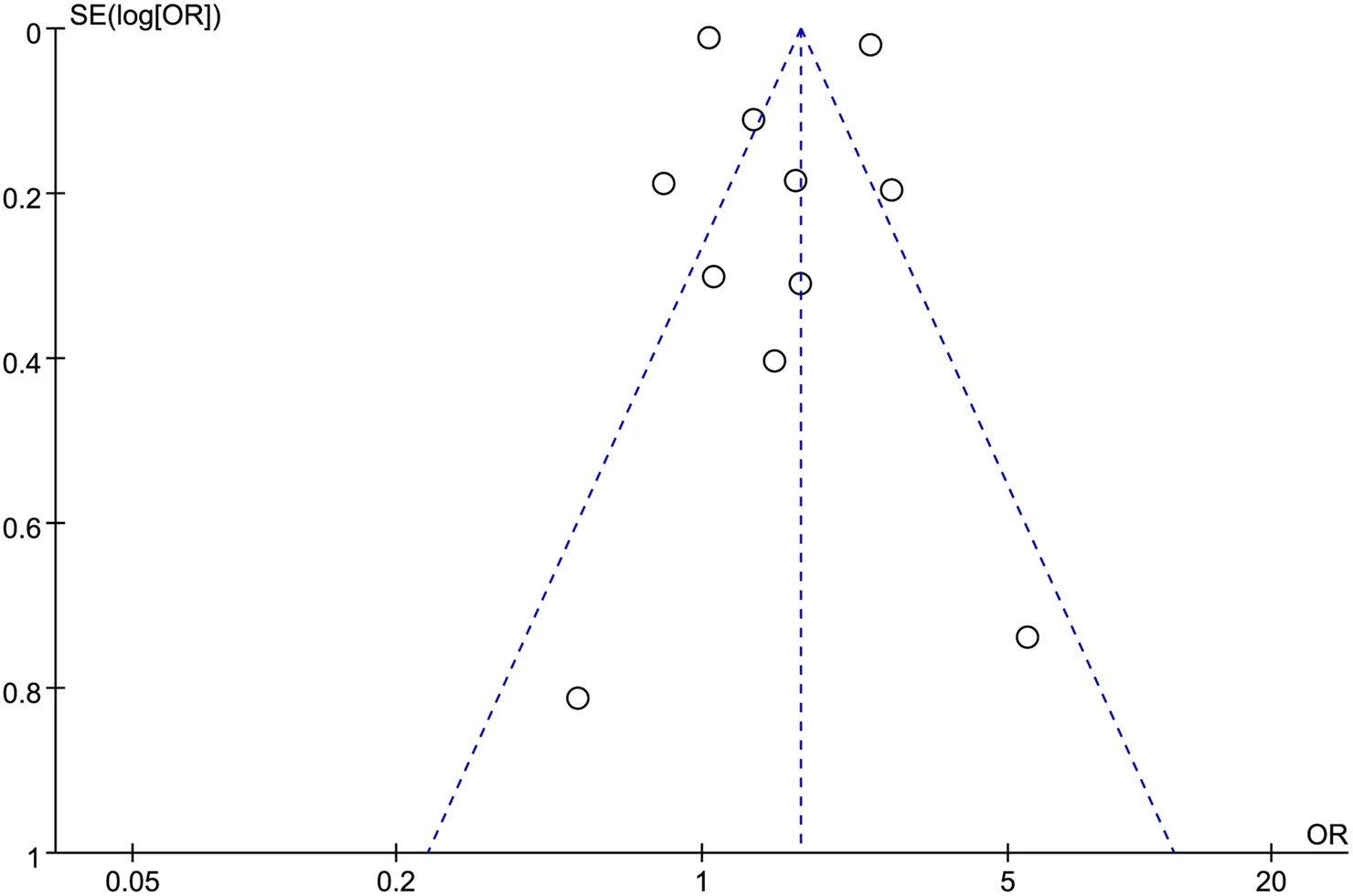
Funnel plot for all-cause mortality.

To assess potential publication bias, a funnel plot was constructed using studies reporting all-cause mortality in this meta-analysis (Figure 6). Visual inspection of the funnel plot did not reveal marked asymmetry; however, interpretation was limited by the small number of included studies. For outcomes with substantial heterogeneity (*I*^2^ > 50%), including all-cause mortality, major amputation, and MACE (Supplementary Figures 2–4), sensitivity analyses were performed due to significant between-study variability. A leave-one-out approach was applied. Sequential exclusion of individual studies did not materially alter the pooled effect estimates for all-cause mortality or major amputation. However, after excluding the study by Zahner et al. (17), the pooled estimate for MACE became more statistically significant (OR = 2.22; 95% CI: 1.28–3.82; *p* < 0.001), with residual heterogeneity (*I*^2^ = 63%).

## Discussion

This meta-analysis suggests that depression in PAD may represent more than a coexisting psychological diagnosis and may serve as a clinically meaningful marker of adverse vascular prognosis. The most consistent findings were observed for limb-related outcomes, with patients with depression showing higher risks of major amputation and MALE. Depression was also associated with MACE, particularly in the time-to-event analysis. In contrast, no statistically significant association was observed between depression and all-cause mortality. Taken together, these findings suggest that depression was associated with poorer clinical outcomes, particularly with respect to limb-related events, while its association with mortality appears less consistent. The association between depression and adverse limb outcomes is biologically and clinically plausible. PAD management depends heavily on sustained self-care, including smoking cessation, exercise therapy, medication adherence, foot surveillance, wound care, and timely follow-up (29–31). Depression can reduce motivation, energy, mobility, and engagement with care, thereby delaying recognition of tissue loss or infection and reducing adherence to limb-preserving treatment (32, 33). In addition, several biological mechanisms may contribute to this association. Depression has been associated with chronic activation of the hypothalamic–pituitary–adrenal axis and sympathetic nervous system, increased systemic inflammation, platelet activation, autonomic dysfunction, and endothelial impairment (34, 35). These alterations may promote atherosclerotic progression, thrombosis, impaired microvascular perfusion, delayed wound healing, and poorer recovery after revascularization. Depression may also indirectly worsen PAD outcomes through reduced physical activity, persistent smoking, unhealthy lifestyle behaviors, and inadequate control of cardiometabolic risk factors. These mechanisms may be especially important in patients with chronic limb-threatening ischemia, in whom even small delays in care or incomplete adherence can translate into major limb loss.

Our findings also add to prior work by suggesting an association between depression and MACE in PAD. This observation was consistent with the broader cardiovascular literature, in which depression has been associated with recurrent events and poorer recovery in coronary disease (36–38). However, interpretation in PAD requires caution. Patients with depression in the included studies had a higher burden of coronary artery disease, diabetes, heart failure, obesity, pulmonary disease, and renal disease. These comorbidities are established contributors to cardiovascular events and may partly mediate or confound the observed association. The more consistent HR-based estimate, together with lower heterogeneity in the time-to-event analysis, suggests that depression may contribute to cumulative cardiovascular risk over time. However, the available observational data cannot determine whether depression is an independent causal factor, a marker of greater overall disease burden, or both. The absence of a statistically significant association with all-cause mortality should not be interpreted as evidence that depression is clinically unimportant in PAD. Mortality is influenced by many competing factors, including age, PAD severity, cancer, infection, frailty, renal disease, and cardiovascular comorbidity. In addition, mortality analyses showed substantial heterogeneity and prediction intervals crossing the null, indicating that the effect may vary across populations and clinical settings. It is possible that depression preferentially affects intermediate outcomes, such as adherence, rehabilitation participation, recurrent symptoms, cardiovascular events, and limb complications, without producing all-cause mortality. Prior observational studies and systematic reviews have examined depression in PAD, including its relationship with mortality, MALE, quality of life, and functional status (28, 30, 39). The contribution of the present study is an updated synthesis with a larger evidence base, simultaneous evaluation of limb, cardiovascular, and mortality outcomes, and incorporation of both event-based and time-to-event estimates when available. This approach helps clarify where the evidence is most consistent, namely, limb outcomes and MACE, and where uncertainty remains, particularly all-cause mortality and the effect of depression severity or treatment.

From a clinical perspective, although our findings do not support depression as an independent predictor of MACE or all-cause mortality in patients with PAD, they highlight a substantial burden of psychosocial distress and cardiometabolic comorbidities in this population. In addition, our results indicate a significantly increased risk of MALE among PAD patients with comorbid depression. Targeted depression screening in comprehensive PAD care may support earlier identification and management, with potential benefits for treatment adherence, functional capacity, rehabilitation participation, and quality of life. However, current evidence is insufficient to determine whether depression screening or treatment directly improves cardiovascular, limb-related, or survival outcomes. Further prospective studies are therefore needed before depression status can be incorporated into prognostic risk stratification or targeted therapeutic decision-making.

## Limitations

Several limitations should be acknowledged. First, the included studies were predominantly retrospective and observational, limiting causal inference and introducing potential confounding bias. Second, despite standardized outcome definitions, substantial heterogeneity persisted, likely due to differences in study design, population characteristics, and depression assessment methods. Third, although no clear publication bias was detected, the assessment remained limited by the small number and type of included studies. Fourth, the lack of data on depression severity and antidepressant treatment restricted subgroup analyses. Fifth, the absence of standardized PAD severity classification precluded stratified analyses and might have contributed to outcome variability. Finally, social determinants of health, including race, socioeconomic status, and access to care, were not adequately addressed.

## Conclusion

In patients with peripheral artery disease, comorbid depression was associated with an increased risk of adverse limb-related outcomes and major adverse cardiovascular events, while no significant association with all-cause mortality was observed. These findings suggests that depression might have influenced clinical outcomes in patients with PAD; however, well-designed prospective studies and randomized controlled trials are needed to further clarify this association.
