# Supplementary material for: Impact of comorbid depression on clinical outcomes in patients with peripheral artery disease: a systematic review and meta-analysis
Source: Front Cardiovasc Med. 2026 Aug 21;13:1854607. doi: 10.3389/fcvm.2026.1854607 (PMC13542983; doi:10.3389/fcvm.2026.1854607)
Supplement: Supplementary file 1 [file Datasheet1.pdf]

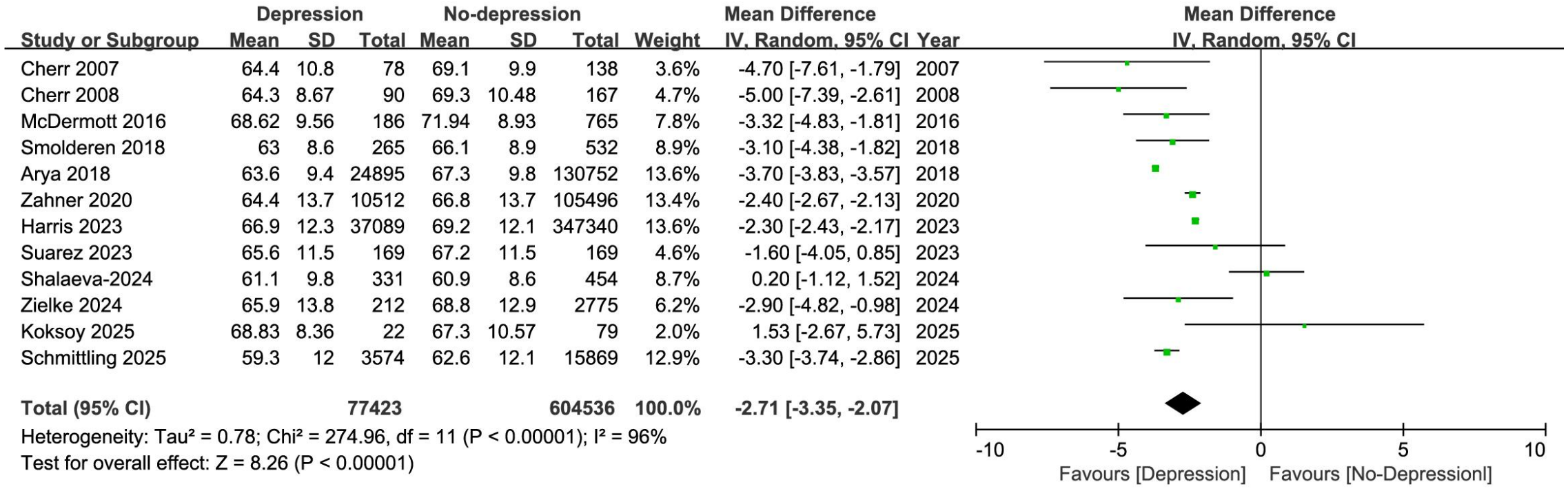


Supplementary Figure 1: Forest plot of the meta-analysis of age between depressed and non-depressed

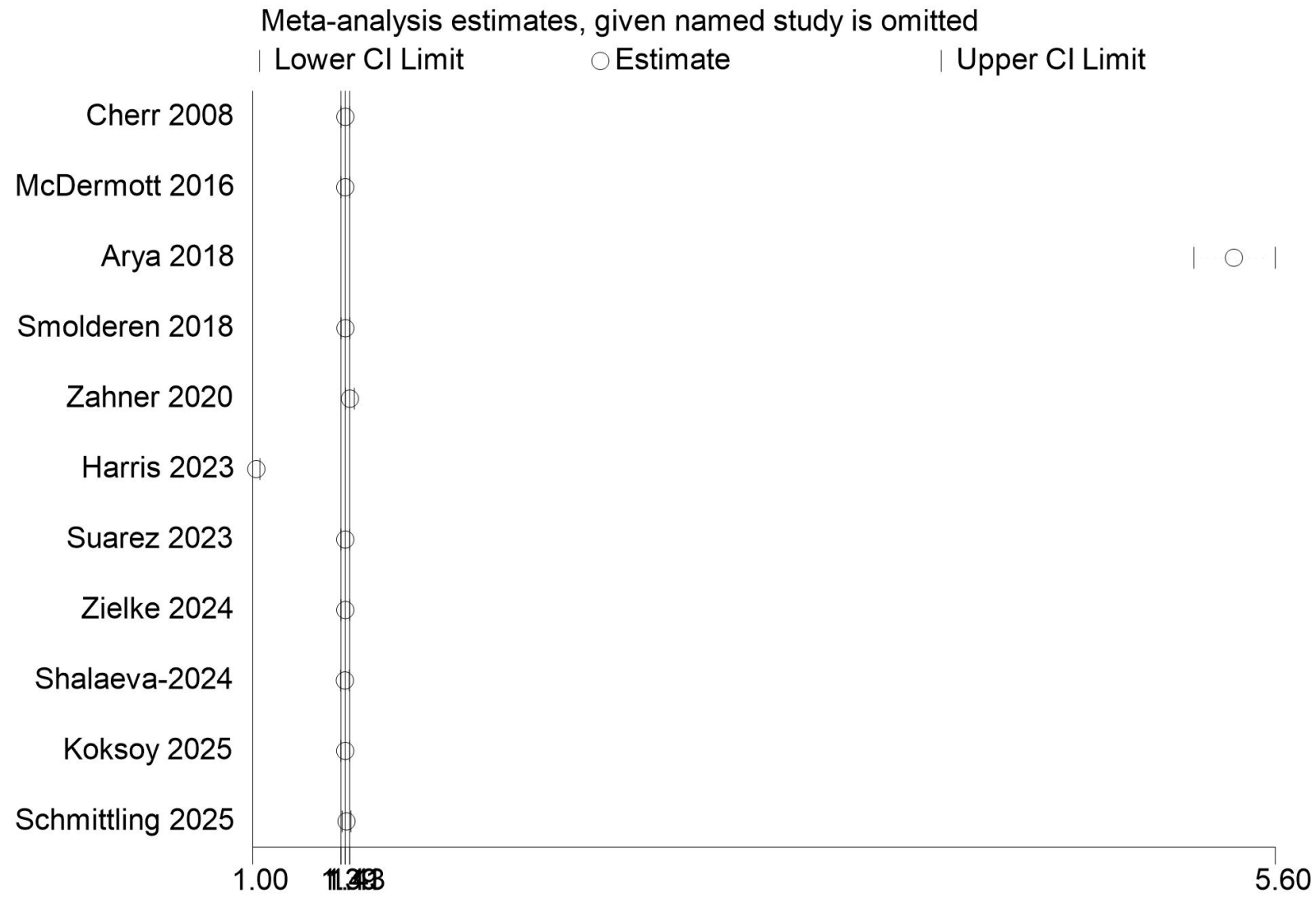


Supplementary Figure 2: Sensitivity analyses were conducted for all-cause mortality

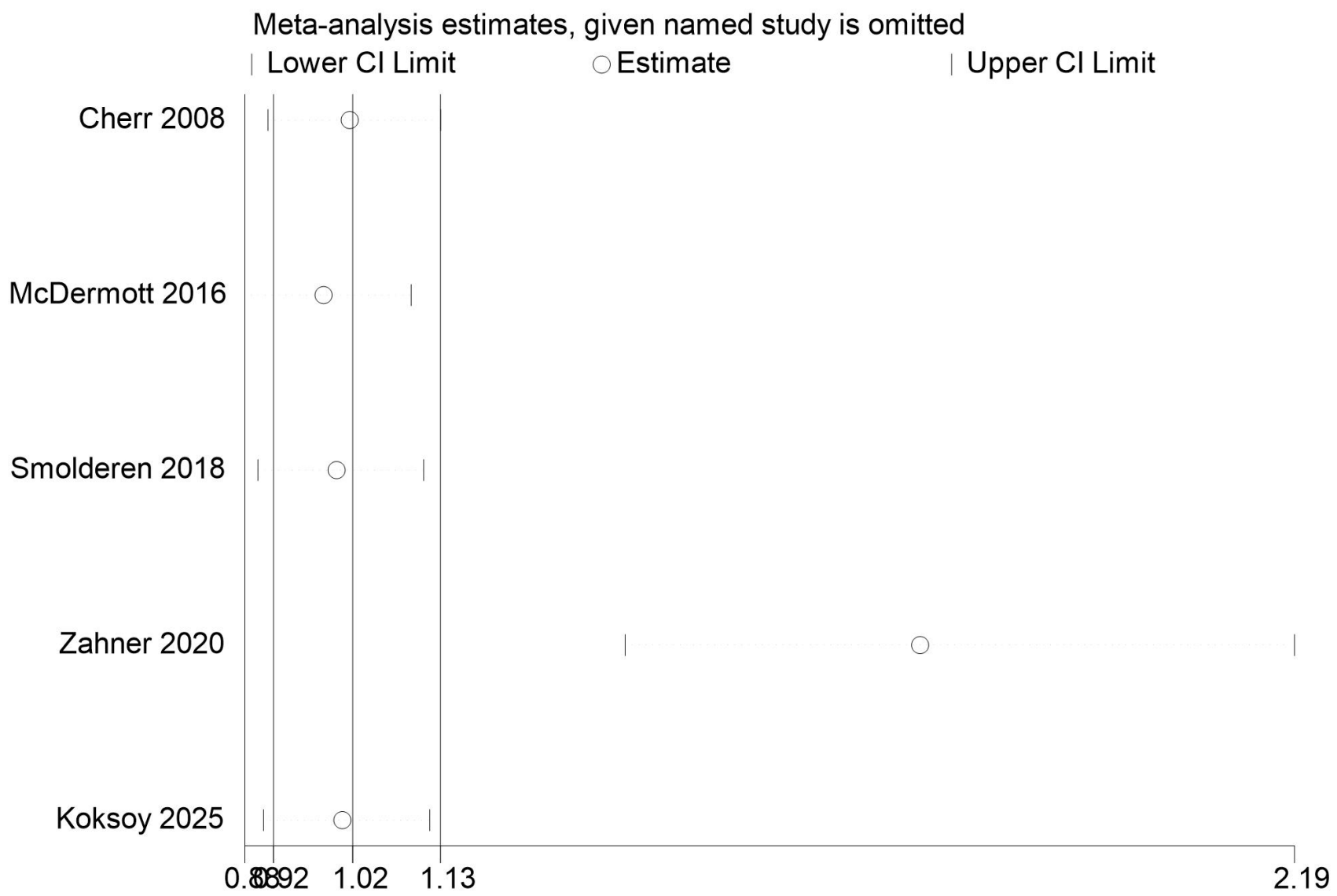


Supplementary Figure 3: Sensitivity analyses were conducted for MACE.

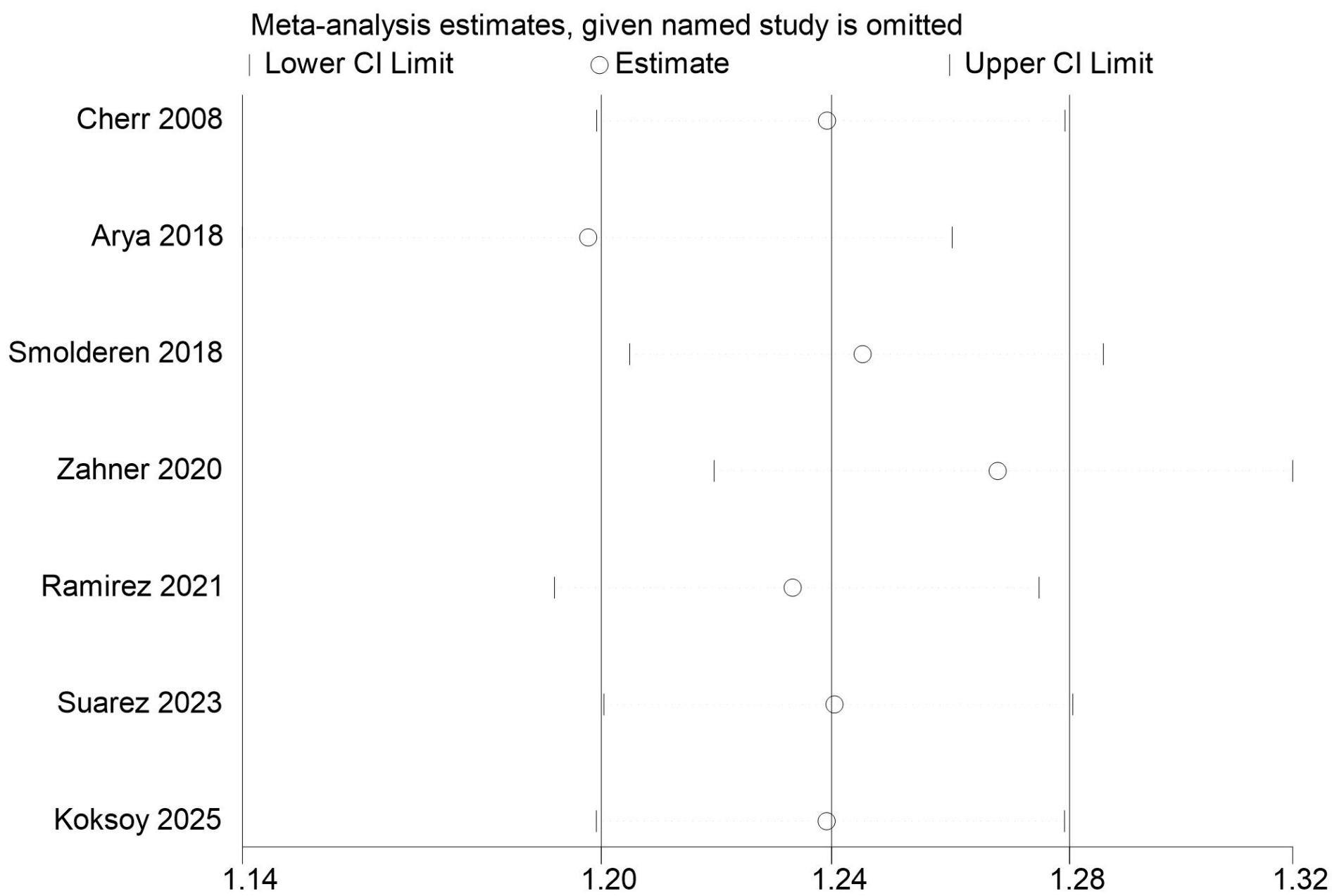


Supplementary Figure 4:Sensitivity analyses were conducted for major amputation
